# Automated periodontal assessment in orthodontic patients: a dual CNN framework

**DOI:** 10.1007/s00784-025-06410-5

**Published:** 2025-06-02

**Authors:** Ebru Yurdakurban, Ali Batuhan Bayırlı, Mehmetcan Uytun, Öznur Suçeken, Onur Karasoy, Osman Özkaraca, Kübra Gülnur Topsakal

**Affiliations:** 1 Faculty of Dentistry, Department of Orthodontics, Muğla Sıtkı Koçman University, Muğla, Turkey; 2 Faculty of Dentistry, Department of Periodontology, Muğla Sıtkı Koçman University, Muğla, Turkey; 3 Faculty of Technology, Information Systems Engineering, Muğla Sıtkı Koçman University, Muğla, Turkey; 4 Gulhane Faculty of Dentistry, Department of Orthodontics, University of Health Sciences, Ankara, Turkey

**Keywords:** Deep learning, Periodontal diseases, Neural networks, Artificial intelligence

## Abstract

**Objective:**

The aim of this study was to develop convolutional neural network (CNN)-based systems to diagnose calculus, plaque, gingival hyperplasia and gingival inflammation in intraoral images from orthodontic patients.

**Materials and methods:**

A dataset of 1,000 lateral and frontal intraoral images from orthodontic patients was used to develop CNN-based models. Periodontology specialists annotated areas of dental calculus, plaque, gingival inflammation, and gingival hyperplasia on the teeth and gingiva. The dataset was divided into training (80%), validation (10%), and test (10%) sets for model development. The YOLOv8 and hybrid U-Net + ResNet50 models were examined. Their performance was evaluated on the basis of accuracy, precision, recall, *F*_1_ score, Tversky loss, intersection over union, mean average precision, Dice coefficient, and Cohen’s kappa.

**Results:**

The mean classification accuracy was 0.96 for the YOLOv8 model and 0.93 for the U-Net + ResNet50 model. On the basis of the Dice coefficient, the models performed best in detecting gingival hyperplasia (YOLOv8: 0.78, U-Net + ResNet50: 0.79) and worst in detecting dental calculus (YOLOv8:0.48, U-Net + ResNet50:0.53). Cohen’s kappa coefficient was highest for classifying gingival hyperplasia (YOLOv8: 0.785, U-Net + ResNet50: 0.790). The precision exceeded 0.72 across all the classifications, with the greatest precision in classifying gingival inflammation.

**Conclusion:**

Deep learning-based systems can serve as decision support tools by offering rapid and objective evaluations of dental calculus, plaque, gingival inflammation, and gingival hyperplasia. Nonetheless, the definitive diagnostic conclusion should be based on the clinician’s specialized expertise and professional judgment.

**Clinical relevance:**

The integration of CNN-based diagnostic models into clinical workflows has the potential to facilitate early periodontal diagnosis and improve accessibility to periodontal assessments in orthodontic patients.

## Introduction

Optimal oral hygiene is considered a key factor for successful orthodontic treatment. Inadequate oral hygiene can lead to periodontal tissue damage, compromising prognosis, prolonging treatment, and resulting in suboptimal outcomes. The long-term stability of functional and aesthetic outcomes depends mainly on the continuous maintenance of periodontal health during and after treatment [1, 2]. Fixed orthodontic appliances, including brackets, bands, and wires, can interfere with effective oral hygiene and dental plaque control. Plaque and calculus accumulation on tooth surfaces and interdental spaces increases the risk of gingival inflammation [3, 4]. Inadequate management of gingival inflammation increases the risk of periodontitis, which is characterized by clinical attachment loss. Consequently, systematic monitoring of gingival health and early detection of pathological changes are critical, particularly in individuals with a history of periodontal disease [5].

Gingivitis is characterized clinically by gingival inflammation, which develops as a host immune response to microbial dental plaque. Common signs include erythema at the gingival margin, bleeding, reduced gingival stippling, increased gingival crevicular fluid, edema, and gingival hyperplasia. Diagnosis involves visually assessing gingival color and morphology, supplemented by clinical periodontal parameters such as the presence of dental plaque and calculus, probing pocket depth, and bleeding [6–8]. When patients have limited access to specialists, intraoral images can serve as a supportive diagnostic tool. Kim et al. reported that intraoral images obtained at various stages of orthodontic treatment enabled the identification of gingivitis through an innovative image analysis approach. Researchers have reported that photographic assessments of gingival papillary health strongly agree with the results of clinical examinations [9].

Recent advances in artificial intelligence (AI) and deep learning (DL) technologies have enabled the development of automatic diagnosis and decision support systems. The convolutional neural network (CNN), a subset of DL techniques, offers successful results in automatic image analysis and classification [10, 11]. Integrating CNN technologies into teledentistry applications enables remote monitoring procedures, facilitating the early detection of periodontal changes in cases where routine check-ups are infrequent [12, 13]. Chau et al. developed an AI model that can detect gingival inflammation using frontal intraoral images and reported that this model could detect gingival inflammation areas with high accuracy [14]. You et al. assessed the accuracy of a DL-based AI model for detecting plaque in primary teeth using intraoral photographs and demonstrated that this model’s automated plaque detection achieved clinically acceptable accuracy compared with evaluations by experienced pediatric dentists [15].

AI-based automated gingivitis diagnostic systems provide significant advantages by ensuring objective and rapid assessments, expediting the diagnostic process, and offering periodontal health insights in cases where access to a dentist is limited [16, 17]. Increasing patients’ awareness of gingivitis and its symptoms to facilitate self-screening may promote timely dental consultations beyond routine orthodontic check-ups. However, accurately detecting gingival inflammation and hyperplasia, as well as plaque and calculus, is crucial for increasing the effectiveness of these systems. Therefore, this study aimed to develop CNN-based models that can diagnose calculus, plaque accumulation, gingival hyperplasia, and gingival inflammation on the basis of intraoral images taken before and after orthodontic treatment and to evaluate their potential for clinical use.

## Methods

### Ethical considerations

This retrospective study received ethical approval from the University of Health Sciences Gulhane Scientific Research Ethics Committee (decision number 2024 − 554) before accessing archival records. All protocols were conducted according to the Declaration of Helsinki. This study was conducted and reported in accordance with the 2015 Standards for Reporting Diagnostic Accuracy Studies (STARD) guidelines [18].

### Data collection and sample selection

A total of 1,000 intraoral images were selected for this study. These images were obtained from clinically and radiographically examined patients and were selected from the archive records of 346 patients treated at the Department of Orthodontics, Gulhane Faculty of Dentistry, University of Health Sciences, between 2018 and 2022. All images were captured using the same digital camera (EOS 1100D; Canon, USA), macro lens (EF 100 mm; Canon, USA), and flash system (MT-24EX Macro Twin Lite; Canon, USA) in fully automatic mode from the right and left lateral and frontal angles. The images were taken at two different times—at the beginning and end of orthodontic treatment—by retracting the lips and cheeks to ensure that only the teeth and gingival tissues were visible.

#### Inclusion and exclusion criteria

This study included patients aged 14–30 years whose intraoral images clearly displayed at least 3 mm of gingiva from the maxillary and mandibular gingival margins, with visible tooth structures. According to the consensus report of the 2017 World Workshop Classification of Periodontal Diseases, only individuals who were diagnosed as periodontally healthy or with gingivitis were included; patients with a diagnosis of periodontitis were excluded [19, 20]. Patients were excluded if they had mucosal diseases not associated with plaque, extensive restorations or caries in the buccal cervical region, periodontal disease-related changes, such as gingival recession, severe clinical attachment loss, alveolar bone resorption, or dark red gingiva without clear melanin pigmentation or signs of inflammation. Additionally, individuals with signs of systemic disease in the oral mucosa or known systemic disease on the basis of anamnesis records were excluded. Finally, intraoral images containing image artifacts that prevented visualization of dental and gingival structures were also excluded from the study.

### Data preparation

The intraoral photographs were annotated by two different periodontologists, each with more than ten years of clinical experience. Initially, M.U. labeled the regions corresponding to the supragingival calculus, dental plaque, gingival hyperplasia, and gingival inflammation. A.B.B. independently reviewed and reevaluated the annotated images. Any discrepancies were resolved through consensus between the two observers to establish reliable ground truth annotations for model training. Cohen’s kappa statistics were used to assess both intra- and interobserver consistency among the raters of the images. The resulting kappa values ranged between 0.80 and 0.92.

Dental plaque is a microbial biofilm on the tooth surface embedded in a matrix of bacterial and salivary polymers [21]. In the intraoral photographs, dental plaque was identified as food debris and deposits located near the gingival margin or interproximal regions. These areas were annotated using a bounding box tool. Supragingival calculus is defined as a mineralized biofilm characterized by various calcium phosphate crystals forming above the free gingival margin [22]. In the intraoral photographs, the supragingival calculus was identified as irregular, yellowish or whitish deposits on the cervical tooth surfaces. These areas were annotated using the bounding box tool. Gingival hyperplasia is the pathological enlargement of gingival tissues and is characterized by an increase in tissue volume; typically, it appears as a red and edematous lesion [23]. The intraoral photographs revealed bulbous or shiny-surfaced gingival contours extending beyond the normal gingival margin, which were annotated using the polygon tool for precise delineation. Gingival inflammation is a reversible pathological process that occurs at the gingival margins due to plaque accumulation and is typically characterized by gingival margins that appear redder and brighter than those of healthy gingiva [24]. The gingival areas showing these visual characteristics were labeled using the polygon tool. Unlabeled areas were separated as background for the CNN model, and five labeled classes were obtained.

Data augmentation techniques increase variability in image datasets, reducing the risk of overfitting and improving the model’s generalizability [25]. Owing to the limited size of the dataset, image transformation and distortion techniques were applied to enhance the performance of the DL models. Geometric transformations such as horizontal flipping, vertical flipping, and image rotation increase the model’s sensitivity to spatial variations, whereas techniques such as random 90° rotation, Gaussian blur, grid distortion, and random brightness and contrast adjustment simulate variations encountered under natural conditions. After the data augmentation process, the total number of images increased to 1,863.

### Training and validation

The dataset was divided into training (*n* = 1491, 80%), validation (*n* = 186, 10%), and test (*n* = 186, 10%) sets for developing the models. Owing to the high computational requirements of the models, the multiple independent randomization method was used to partition the datasets. Object detection architectures based on both one-stage and two-stage detector models were used. Single-stage detectors perform object localization and classification within a single CNN, enabling simultaneous execution. In contrast, two-stage detectors follow a sequential process, first generating region proposals and then refining bounding box coordinates through classification [26].

**Fig. 1:**
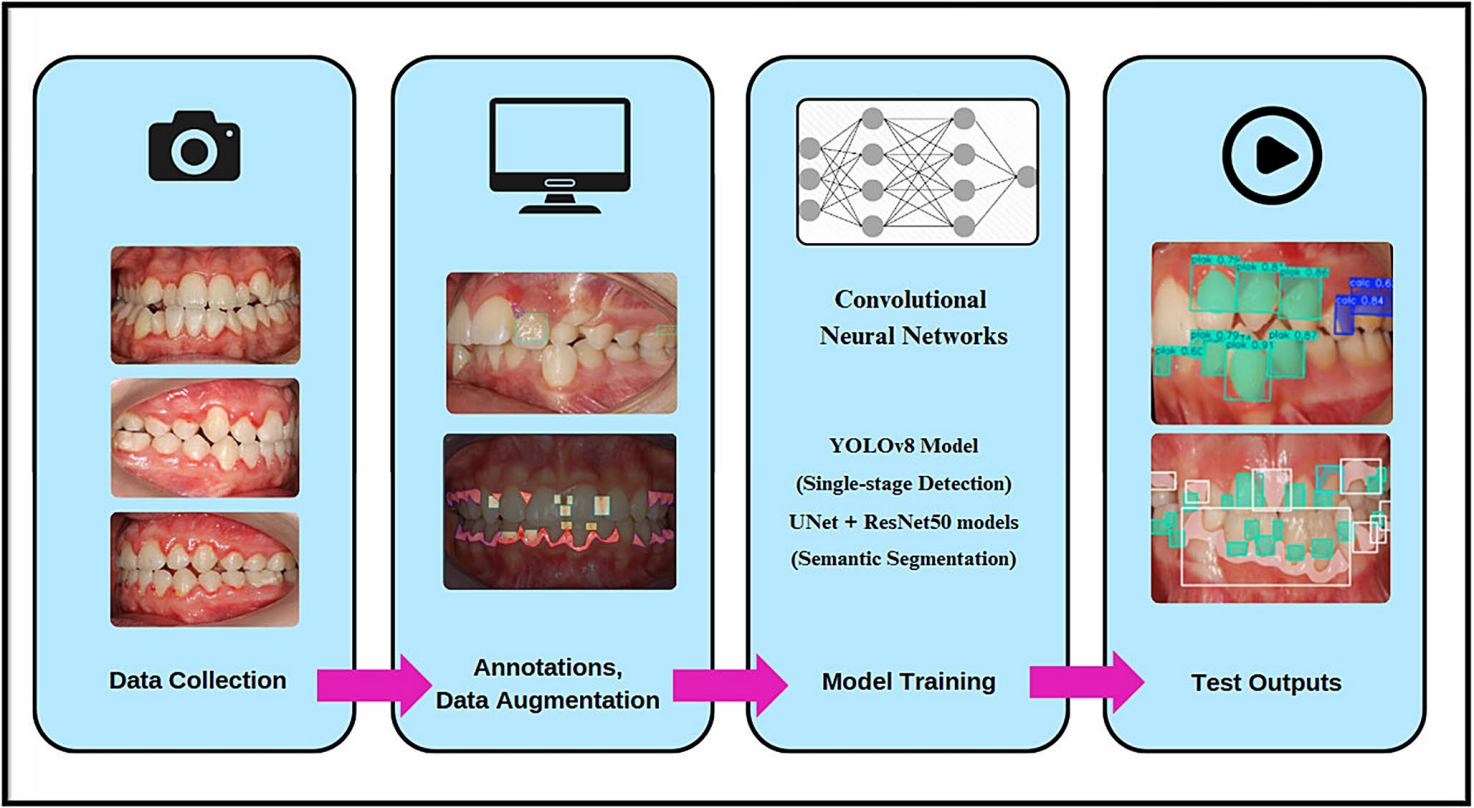
Study flowchart

This study used version eight of the you only look once model (YOLOv8) to represent single-stage detection architectures and the U-Net + ResNet50 hybrid model to represent two-stage detection architectures. The performance of all the models was compared on a Windows 10 system equipped with a T4 GPU (NVIDIA, USA). The study workflow is shown in Fig. 1.

### YOLOv8

**Fig. 2:**
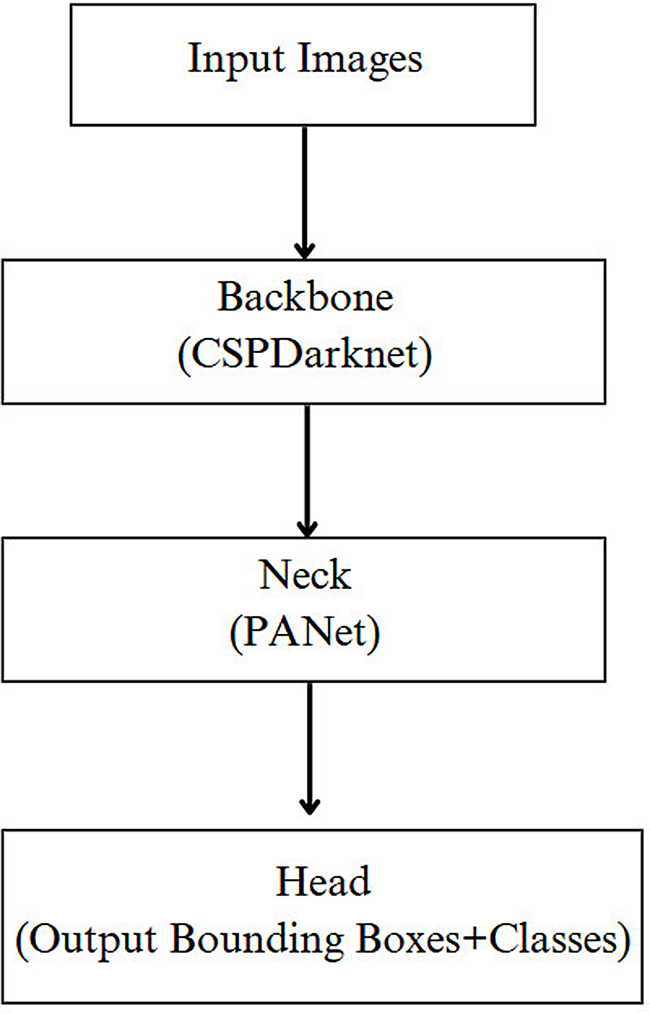
YOLOv8 model architecture

This study used the YOLOv8 object detection model, which is designed to perform fast and accurate localization and classification of multiple objects in images (Fig. 2). The primary reason for selecting this algorithm was its high performance in detecting small and localized features, such as dental calculus, plaque, and inflamed regions, in high-resolution intraoral images. The YOLO model uses a single-stage architecture, distinguishing it from conventional two-stage object detection methods by processing input images through a CNN that simultaneously predicts bounding boxes and class labels. Conversely, two-stage models (such as R-CNN and Fast R-CNN) first generate potential object regions and then classify them, which is computationally intensive and time-consuming [27].

**Fig. 3:**
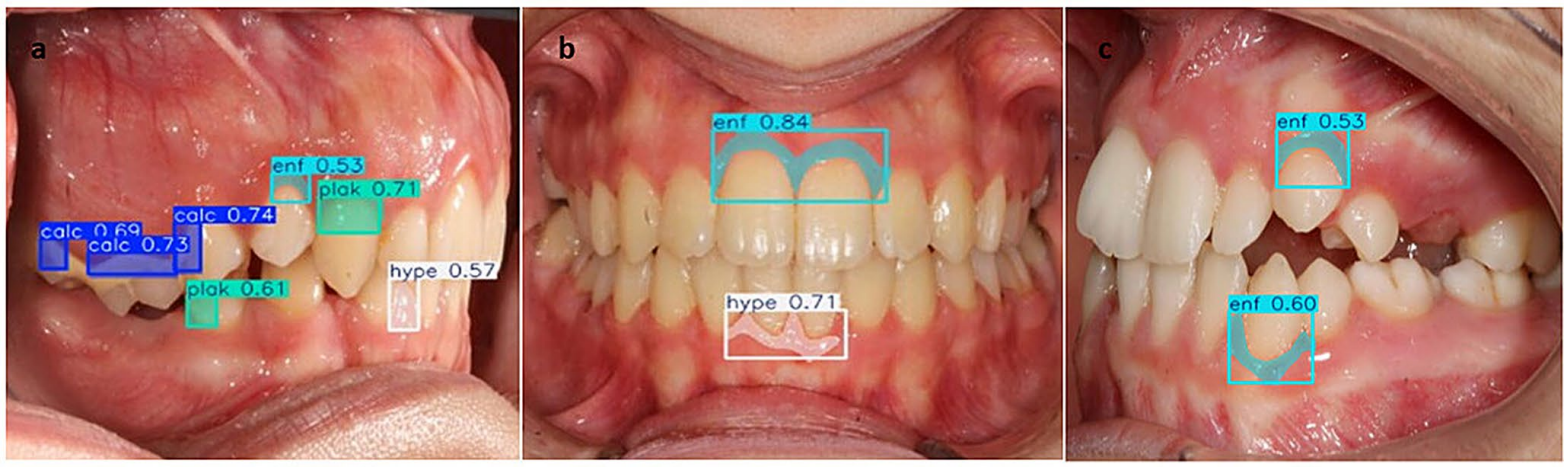
Intraoral images displaying YOLOv8 model predictions. (**a**–**c**) Bounding boxes represent detected regions: blue indicates dental calculus, green indicates dental plaque, cyan indicates gingival inflammation, and pink indicates gingival hyperplasia

The single-pass design of YOLO supports millisecond-level latency, making it particularly well suited for clinical environments where timely analysis is crucial. YOLO divides each image into a grid and estimates bounding boxes and class probabilities for each cell, allowing precise detection of small, confined anomalies (Fig. 3). Furthermore, YOLOv8 incorporates architectural enhancements such as the cross-stage partial network (CSPNet) and path aggregation network (PANet), which improve feature extraction while maintaining a lightweight structure. As a result, the model can be deployed even on systems with limited memory capacity without compromising accuracy [28, 29].

### U-Net + ResNet50: a hybrid model for semantic segmentation

Semantic segmentation is a computer vision task that involves classifying each pixel in an image into a specific category. U-Net was selected for its ability to learn effectively from limited data and to generate high-resolution outputs that can accurately delineate fine structures, which is particularly important for identifying localized anomalies. ResNet50 was preferred over U-Net’s original encoder due to its residual connections, which enable deeper network training and mitigate the vanishing gradient problem, thereby improving representational capacity. This approach also strengthens generalizability and improves model performance in medical imaging tasks that typically involve small, annotated datasets.

**Fig. 4:**
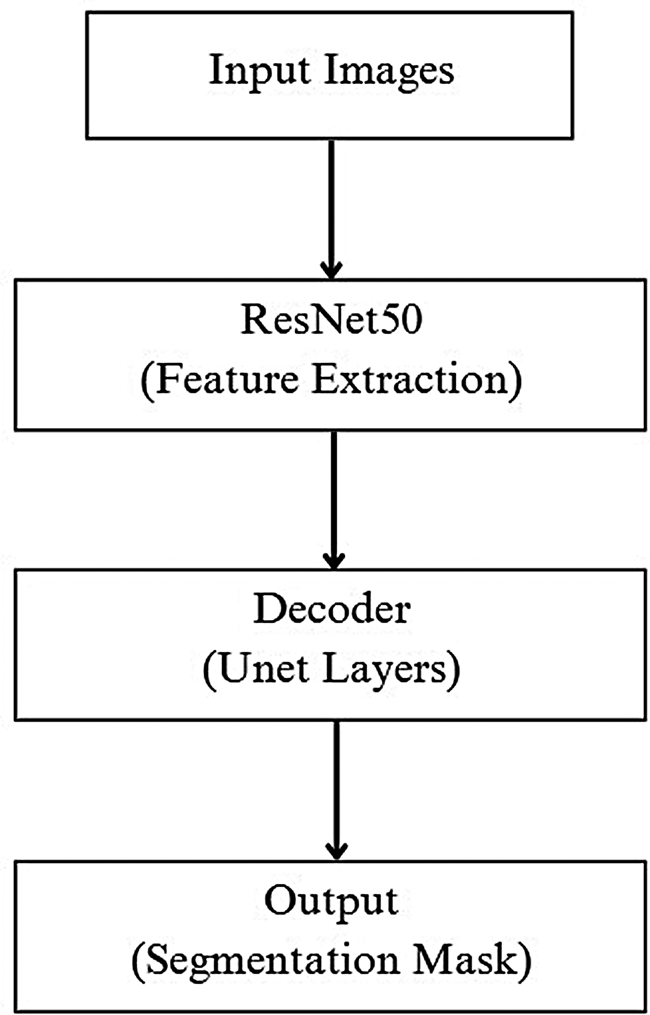
U-Net + ResNet50 model architecture

In this study, segmentation was performed using a hybrid deep learning architecture that integrates the U-Net and ResNet50 models (Fig. 4). In the technical framework of this hybrid structure, ResNet50 was applied to the encoder segment to extract high abstraction feature maps from the input image. These features are then passed to the traditional decoder component of U-Net. Here, upsampling and skip connections are used to reconstruct segmentation maps that closely preserved the original resolution. Thus, the model has both a deep encoder that can capture the global context and a powerful decoder that can preserve fine details. Furthermore, the pretrained weights of ResNet50 contribute to faster and more stable model learning through transfer learning. This hybrid structure significantly improves the generalizability and accuracy of the model, especially in the field of medical imaging with limited labeled data.

 [30–33].

### Evaluation metrics

Model performance was assessed using the following metrics: accuracy, precision, recall, *F*_1_ score, Tversky loss, intersection over union (IoU), mean average precision (mAP), Dice coefficient, Cohen’s kappa (*κ*) and confidence interval (CI).


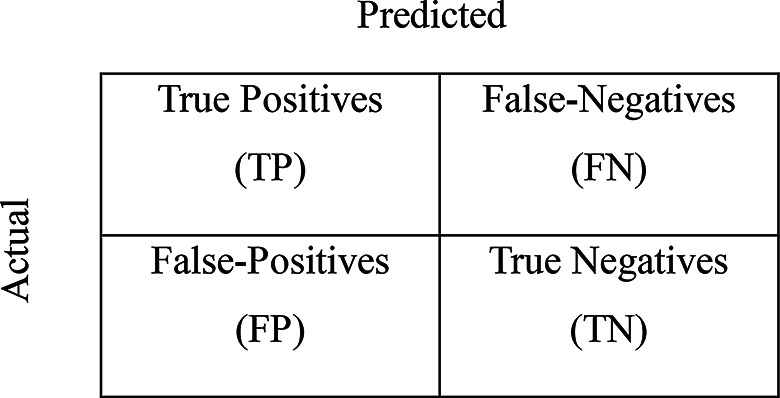


Accuracy is defined as the ratio of correctly classified true positive (TP) and true negative (TN) predictions to the total number of predictions (TP, TN, false-positive [FP], and false-negative [FN]) [34]:

$$\:Accuracy\:=\:\frac{TP+TN}{TP+TN+FP+FN}$$

Precision is defined as the proportion of TP predictions among all instances classified as positive by the model. It measures the model’s ability to correctly identify positive cases while accounting for FP predictions [34]:

$$\:Precision=\:\frac{TP}{TP+FP}$$

Recall is defined as the proportion of actual positive instances correctly identified by the model. It measures the model’s sensitivity in detecting positive cases while considering the impact of FN predictions [34]:

$$\:Recall=\:\frac{TP}{TP+FN}$$

The *F*_1_ score is defined as the harmonic mean of precision and recall, providing a single measure that balances both metrics. It is particularly useful for evaluating model performance based on imbalanced datasets, as it accounts for both FP and FN predictions. The *F*_1_ score is often preferred over accuracy when the class distribution is uneven [34]:

$$\:{F}_{1}=2\times\:\frac{precision\times\:recall}{precision+recall}$$

The mAP is a performance metric that measures the overall model accuracy by averaging the individual average precision scores across multiple classes or objects. It is computed as the mean area under the precision‒recall curve for each class, providing a comprehensive assessment of both the model’s precision and recall [35]:

$$mAP = {1 \over {\left| {classes} \right|}}\sum\limits_{c\> \in \>\>classes} {{{{{\left| {TP} \right|}_c}} \over {{{\left| {FP} \right|}_c} + \>{{\left| {TP} \right|}_c}}}}$$

The IoU is a metric used to measure the degree of overlap between the model’s prediction and the ground truth. It is calculated as the ratio of the intersection area to the union area of the predicted and actual regions and provides a quantitative assessment of localization accuracy [36]:

$$\:IoU=\:\frac{TP}{TP+FP+FN}$$

Tversky loss is a loss function used in image segmentation tasks, particularly in medical image analysis and computer vision. It measures the difference between two sets, such as the predicted and ground truth binary masks used in segmentation. The function includes weighting parameters that control the balance between FPs (*α*) and FNs (*β*), allowing for adaptive weighting on the basis of task-specific requirements [37]:

$$\:Tl=\frac{TP}{TP+\:\alpha\:FN+\:\beta\:FP}$$

The Dice coefficient is a metric used extensively in medical image processing and for evaluating segmentation accuracy. It measures the similarity between two sets by measuring the overlap between the predicted and ground truth bounding boxes [38]:

$$\:Dice=\:\frac{2\times\:TP}{(TP+FP)+(TP+FN)}$$

Cohen’s *κ* removes the agreement between the raters from the agreement due to chance and reveals the true level of agreement with the results. It measures the agreement of the predicted classes with the actual classes [39]:

$$\:N=TP+TN+FP+FN$$

$$\kappa = {{{{TP + TN} \over N} - \left[ {{{\left( {TP + FP} \right) \cdot \left( {TP + FN} \right)} \over {{N^2}}} + {{\left( {TN + FN} \right) \cdot \left( {TN + FP} \right)} \over {{N^2}}}} \right]} \over {1 - \left[ {{{\left( {TP + FP} \right) \cdot \left( {TP + FN} \right)} \over {{N^2}}} + {{\left( {TN + FN} \right) \cdot \left( {TN + FP} \right)} \over {{N^2}}}} \right]}}$$

The confidence interval is a measure that provides information about the accuracy of a statistical estimate. In this study, the CI corresponding to the 95% confidence level was calculated using the following formula.

$$CI = \bar x \pm 1.96 \times {s \over {\sqrt n }}$$

Here, $\bar x$ is the sample mean, s is the sample standard deviation, n is the number of trials, and t_(α\/2,n-1) is the critical value obtained from the t distribution. When the confidence level is 95%, α is 0.05. This value affects the width of the confidence interval. S is the sample standard deviation. It measures how much the data deviate from the sample mean [40].

## Results

**Fig. 5:**
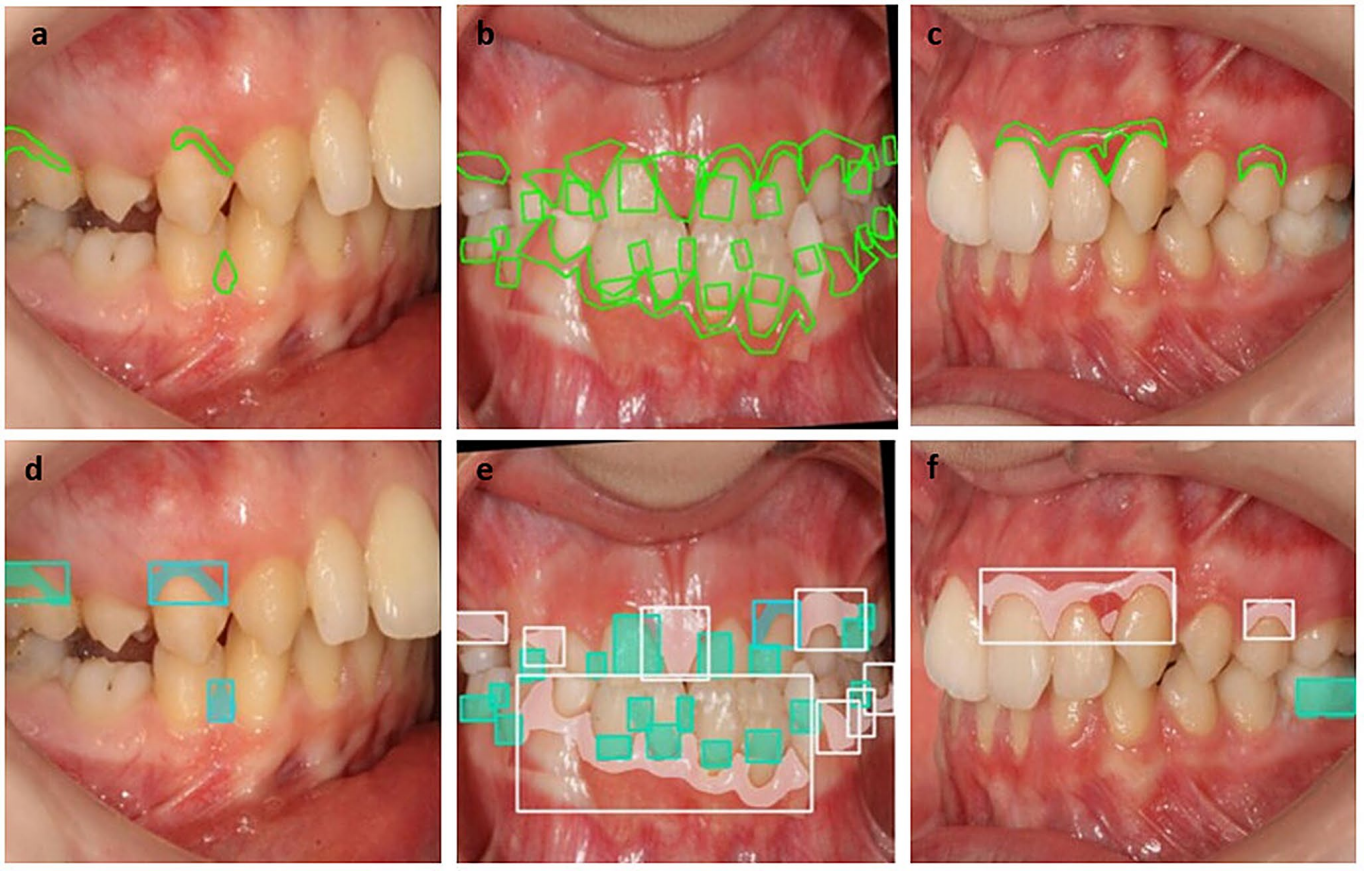
Intraoral images showing expert annotations and U-Net + ResNet50 model predictions. (**a-c**) Green outlines represent regions manually labeled by experts. (**d**-**f**) Model predictions: pink indicates gingival hyperplasia, green indicates plaque, and cyan indicates gingival inflammation

In this study, 1,000 intraoral images were annotated, and predictions were output by the models (Figs. 3 and 5). The performance metrics of the models developed using the YOLOv8 and U-Net + ResNet50 architectures for detecting calculus, plaque, gingival inflammation, and gingival hyperplasia are presented in Tables 1 and 2. Both models achieved high accuracy, with mean accuracies of 0.90–0.97 in the classification tasks. The mean accuracy across all classifications was 0.961 ± 0.006 for the YOLOv8 model and 0.938 ± 0.027 for the U-Net + ResNet50 model. The greatest accuracy was obtained for dental calculus with the YOLOv8 model (0.969) and gingival hyperplasia with the U-Net + ResNet50 model (0.970).

**Table 1:** Performance evaluation results using the YOLOv8 model for the detection of dental calculus, dental plaque, gingival hyperplasia, and gingival inflammation based on the testing set

YOLOv8	Accuracy	Dice Coefficient	Cohen’s Kappa	Twersky Loss	mAP	Precision	F1-score	IoU	Recall
Dental Calculus	0.969	0.480	0.490	0.090	0.570	0.800	0.570	0.350	0.350
Gingival Inflammation	0.950	0.640	0.620	0.110	0.600	0.765	0.670	0.440	0.450
Gingival Hyperplasia	0.960	0.785	0.785	0.080	0.650	0.850	0.780	0.590	0.700
Dental Plaque	0.951	0.540	0.530	0.125	0.590	0.720	0.500	0.370	0.400
Mean ± SD	0.961 ± 0.006	0.612 ± 0.440	0.606 ± 0.461	0.112 ± 0.041	0.603 ± 0.009	0.784 ± 0.010	0.630 ± 0.007	0.438 ± 0.011	0.475 ± 0.007
95% CI	[0.9566,0.9654]	[0.5805,0.6435]	[0.5730,0.6390]	[0.0822,0.1418]	[0.5962,0.6098]	[0.7762,0.7918]	[0.6244,0.6356]	[0.4301,0.4459]	[0.4695,0.4805]

SD: standard deviation, CI: confidence interval, mAP: mean average precision, IoU: intersection over the union

**Table 2:** Performance evaluation results using the U-Net + ResNet50 hybrid model for the detection of dental calculus, dental plaque, gingival hyperplasia, and gingival inflammation based on the testing set

U-Net + ResNet50	Accuracy	Dice Coefficient	Cohen’s Kappa	Twersky Loss	mAP	Precision	F1-score	IoU	Recall
Dental Calculus	0.920	0.530	0.550	0.140	0.560	0.730	0.570	0.350	0.350
Gingiva Inflammation	0.960	0.700	0.670	0.090	0.580	0.810	0.600	0.460	0.470
Gingival Hyperplasia	0.970	0.790	0.790	0.065	0.640	0.850	0.790	0.600	0.750
Dental Plaque	0.900	0.550	0.500	0.110	0.580	0.740	0.600	0.420	0.420
Mean ± SD	0.938 ± 0.027	0.645 ± 0.046	0.628 ± 0.082	0.101 ± 0.045	0.590 ± 0.056	0.783 ± 0.056	0.640 ± 0.046	0.458 ± 0.031	0.498 ± 0.067
95% CI	[0.9182,0.9578]	[0.6119,0.6781]	[0.5688,0.6872]	[0.0686,0.1334]	[0.5494,0.6306]	[0.7424,0.8236]	[0.6069,0.6731]	[0.4355,0.4805]	[0.4496,0.5464]

SD: standard deviation, CI: confidence interval, mAP: mean average precision, IoU: intersection over the union

**Fig. 6:**
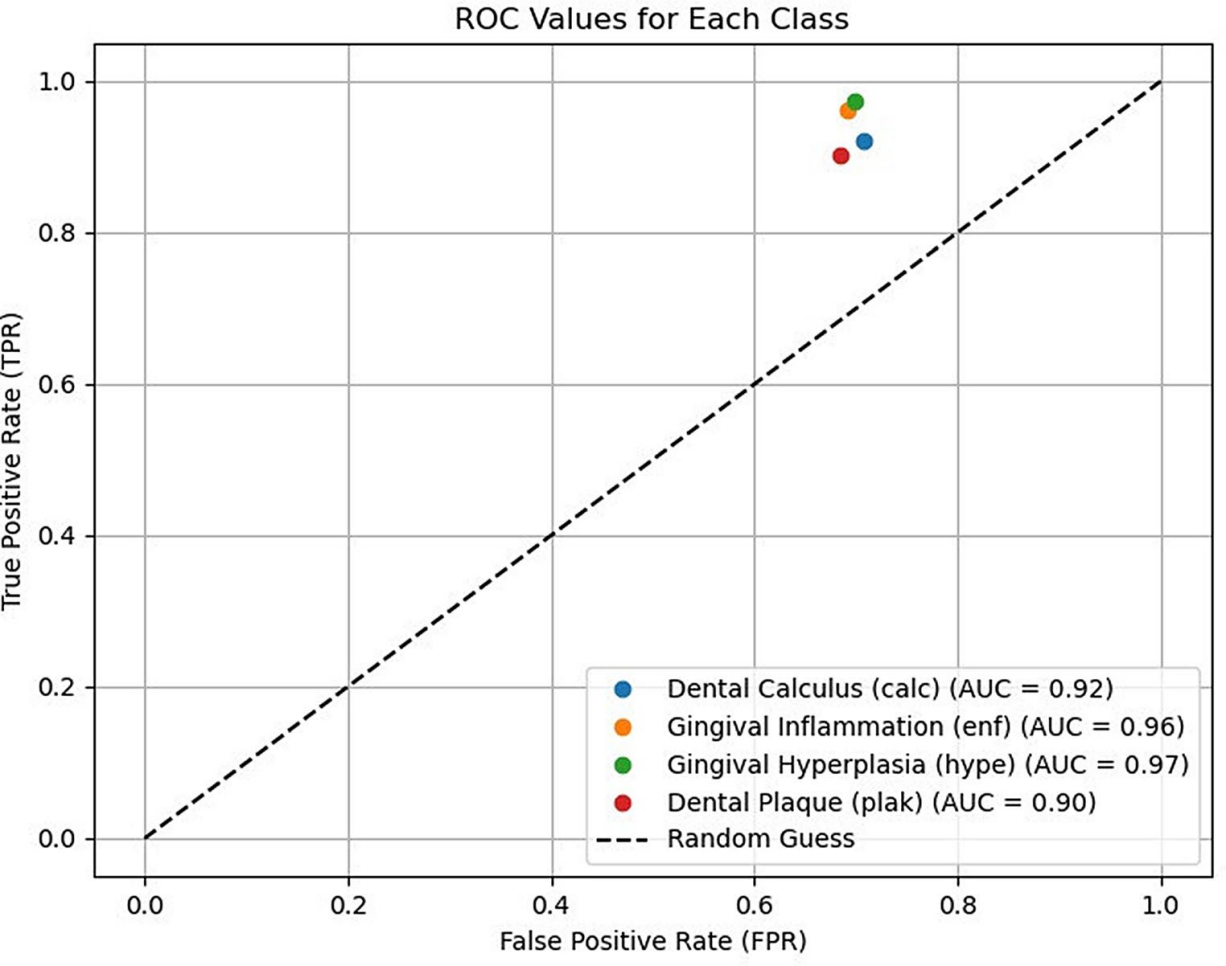
AUC values showing the average classification performance of the YOLOv8 and U-Net + ResNet50 models

Receiver operating characteristic (ROC) analysis demonstrated the mean classification performance of the YOLOv8 and U-Net + ResNet50 models in detecting periodontal conditions, with an area under the ROC curve (AUC) exceeding 0.90, indicating high diagnostic accuracy. Discrimination was greatest for gingival hyperplasia (AUC = 0.97) and inflammation (AUC = 0.96) and was slightly lower but consistent for dental calculus (AUC = 0.92) and plaque (AUC = 0.90). The models’ separation from the random chance line validated their reliability in distinguishing pathological and healthy tissue (Fig. 6).

The Dice coefficient indicated that the models performed best in detecting gingival hyperplasia (YOLOv8: 0.785, U-Net + ResNet50: 0.790) and worst in detecting dental calculus (YOLOv8: 0.480, U-Net + ResNet50: 0.530). Cohen’s κ was highest in gingival hyperplasia classification (YOLOv8: 0.785, U-Net + ResNet50: 0.790), indicating a strong correlation between model predictions and expert assessments. The mean Twersky loss was 0.112 for the YOLOv8 model and 0.101 for the U-Net + ResNet50 model, indicating that both models effectively minimized classification errors.

The precision was above 0.72 for all classes and was greatest for the gingival inflammation classification (0.850). Conversely, the recall was less than 0.50 in all classes except for gingival hyperplasia detection. The mAP was highest for gingival hyperplasia detection (YOLOv8: 0.650, U-Net + ResNet50: 0.640) and lower for calculus and dental plaque detection (YOLOv8: 0.570, U-Net + ResNet50: 0.560, YOLOv8: 0.590, U-Net + ResNet50: 0.580).

The YOLOv8 and U-Net + ResNet50 models demonstrated consistent and reliable performance in the detection and segmentation of dental calculus, dental plaque, gingival hyperplasia, and gingival inflammation on the basis of the 95% confidence intervals. The mean accuracy was 0.961 ± 0.0044 (95% CI: [0.9566, 0.9654]) for YOLOv8 and 0.938 ± 0.0198 (95% CI: [0.9182, 0.9578]) for U-Net + ResNet50. The Dice coefficients were 0.612 ± 0.0315 (95% CI: [0.5805, 0.6435]) and 0.645 ± 0.0331 (95% CI: [0.6119, 0.6781]), respectively. The Cohen’s kappa values were 0.606 ± 0.0330 (95% CI: [0.5730, 0.6390]) for YOLOv8 and 0.628 ± 0.0592 (95% CI: [0.5688, 0.6872]) for U-Net + ResNet50. The F1 scores were 0.630 ± 0.0056 (95% CI: [0.6244, 0.6356]) and 0.640 ± 0.0331 (95% CI: [0.6069, 0.6731]), respectively (Tables 1 and 2).

## Discussion

In various fields of dentistry, new AI-based systems have been developed for clinical decision support, integrating photographs and radiographs. These tools perform tasks such as automated cephalometric analysis, periodontal bone loss detection, and mucosal lesion prediction from radiographs, as well as caries detection and prediction of treatment results from photographs [41–43]. Assessing periodontal health is critical in determining treatment suitability before orthodontic intervention and maintaining permanent aesthetic and functional results after treatment [1]. CNN-based AI models can facilitate objective and rapid evaluation of periodontal problems, including plaque accumulation, calculus formation, and gingival inflammation [10]. To the best of our knowledge, previous studies have focused predominantly on evaluating gingivitis and a limited set of parameters using intraoral photographs captured primarily from frontal angles. Unlike these prior approaches, in this study, CNN-based models were developed to detect dental calculus, dental plaque, gingival hyperplasia, and gingival inflammation simultaneously from intraoral images captured from both frontal and lateral angles, which are routinely used in orthodontic records, and assess their clinical applicability.

Different DL models demonstrate superior performance in specific tasks, such as object detection and semantic segmentation, on the basis of their architectural characteristics and learning capacities. These variations arise from differences in data processing methods, feature extraction capabilities, and learning strategies. Unlike previous studies in the literature, we used a hybrid encoder–decoder architecture for semantic segmentation, integrating U-Net and ResNet50. This integration led to high accuracy and precision in identifying gingival hyperplasia and inflammation through effective delineation of relevant regions. For object detection, the YOLOv8 model was employed. This model demonstrated superior performance in detecting dental calculus and plaque. The mean accuracy was close to the maximum of 1.0 for both models, indicating that they achieved clinically acceptable accuracy for assessing dental calculus, dental plaque, gingival inflammation, and gingival hyperplasia. The precision of these models exceeded 0.72, suggesting that both models can accurately identify periodontal conditions with a low FP rate.

Li et al. [44] developed a CNN model on the basis of multitask learning using 3,932 intraoral images taken with different devices, including smartphones and digital cameras. Their hybrid model, which includes both classification and object detection approaches, achieved high AUC values for gingivitis (87.11%), dental calculus (80.11%), and soft deposits (78.57%), indicating robust classification performance. The performance of the object detection approach in their study aligns with the high accuracy observed with the YOLOv8 model for dental plaque and calculus detection in our study. You et al. [15] employed the DeepLabV3 + model, which performs semantic segmentation by analyzing the class assignment of each pixel. It is particularly effective in delineating soft tissues with indistinct boundaries. They reported that the DeepLabV3 + model achieved a mean IoU comparable to that of expert evaluations in dental plaque segmentation. Similarly, Li et al. [45] compared different CNN architectures, including ResNet, GoogLeNet, AlexNet, and VGG. Among them, ResNet performed best, achieving an AUC of 97%. Aykol-Sahin et al. [46] utilized the ResNet50, ResNet18, MobileNetV2, and DeepLabV3 + models to detect and measure oral keratinized gingiva. The ResNet50 model demonstrated the greatest accuracy in identifying keratinized gingiva (91.4%). They suggested that CNN-based models could serve as assistive tools for clinicians in image-based diagnosis.

Consistent with findings from previous studies, our study demonstrated that the U-Net + ResNet50 model achieved greater accuracy and precision in detecting gingival hyperplasia and inflammation. These results indicate that segmentation-based models are more effective in identifying periodontal conditions in soft tissues with indistinct boundaries, whereas object detection-based models, such as YOLOv8, perform better in distinguishing hard tissue structures such as dental calculus and plaque, yielding results that align more closely with expert evaluations. This contrast can be attributed to both the morphological characteristics of the conditions and the model architectures. Dental calculus and plaque are sharply localized and visually distinct, favoring detection by bounding boxes, whereas hyperplasia and inflammation require fine-grained pixelwise analysis owing to their diffuse appearance. Accordingly, our models achieved higher detection accuracy for dental calculus and plaque using YOLOv8 and better segmentation performance for hyperplasia and inflammation with U-Net + ResNet50.

The performance of CNN-based image classification models is determined not only by their architectural design but also by factors such as data augmentation strategies, dataset size, and class balance. Luo et al. [47] investigated the impact of dataset size on CNN-based classification performance and demonstrated that models trained based on larger datasets generally achieve greater accuracy. Our study applied data augmentation, increasing the total number of images to 1,863, and the mean accuracy for both models was close to the maximum of 1.0. Alalharith et al. [48] developed a DL-based model for the automated identification of teeth and early gingivitis symptoms in orthodontic patients using 134 intraoral images. Their model achieved 100% accuracy in tooth identification, with accuracies in distinguishing inflamed and noninflamed gingival areas of 78.46% and 75.79%, respectively. In our study, the greater mean accuracy obtained for gingival inflammation classification can be attributed not only to the use of hybrid segmentation architectures but also to the larger dataset, which likely contributed to enhanced model performance.

Luo et al. [47] emphasized that the negative impact of dataset imbalance on model performance becomes more pronounced as the number of classification categories increases. The mean mAP, Dice coefficient, and Cohen’s κ were generally low, except for those of the gingival hyperplasia classification. This result can be considered a consequence of the unbalanced distribution within the dataset. Higher means were observed for all evaluation metrics in the gingival hyperplasia class, which contained more samples, whereas worse performance was observed for classes with fewer samples. In addition, the models not only detected the presence of periodontal problems but also performed localization tasks. However, the low mean mAP suggests that, despite correctly classifying periodontal conditions, the models may exhibit inaccuracies in localizing specific regions on the teeth and gingival structures. Therefore, clinicians should consider potential localization errors during periodontal assessments.

The proposed model demonstrated promising results in supporting periodontal assessment and holds potential for real-world applications. When integrated into teledentistry platforms, it could provide automated documentation, particularly in remote or underserved populations with limited access to dental care. This capability could support early diagnosis, helping to prevent progression to periodontitis and irreversible tissue damage. This approach may improve the accuracy of periodontal assessments in environments lacking periodontal specialists or sufficient clinical expertise. Additionally, it may reduce chair time, offer workforce and time savings, and serve as an effective aid in dental education and training. Although newly introduced artificial intelligence-based systems offer numerous advantages in health care, rigorous validation and resolution of existing limitations are essential before their full integration into hospital and clinical workflows.

Investigating three-dimensional segmentation with hybrid architectures that integrate two-dimensional intraoral images may improve the diagnostic accuracy of the model by allowing a more detailed analysis of periodontal structures. The inherently complex 3D anatomy of periodontal tissues, particularly in conditions such as gingival hyperplasia, supports the need for volumetric assessment methods. Li et al. reported that intraoral scanners and physical models yield more reliable outcomes than photographs do in periodontal phenotype evaluation [49]. Yang et al. demonstrated that 3D AI-based segmentation methods for measuring gingival thickness are highly correlated with manual measurements [50]. However, AI systems trained exclusively with intraoral photographs remain limited to two-dimensional interpretation. Integrating 3D data and hybrid segmentation approaches may increase the precision of automated detection, especially for volumetric soft tissue conditions such as gingival hyperplasia.

Our study had several limitations. First, it uses a limited number of intraoral images to train the developed models. Increasing the dataset size could enhance performance and improve applicability across different cases. As the dataset was derived from a single institution, the generalizability of the model to broader populations may be limited. The inclusion of intraoral images of patients from diverse ethnic backgrounds may increase the inclusiveness of the model. Second, only images captured from frontal and lateral angles were included. The inclusion of images taken from different angles may improve model performance and provide a more comprehensive and reliable periodontal assessment. Additionally, image variability was constrained by consistent lighting and camera conditions, which may affect the model’s robustness in more heterogeneous settings. Future studies should consider the use of larger, multicenter datasets with a wider range of image sources and patient diversity to improve diagnostic performance and clinical applicability.

Our study included images that did not contain fixed orthodontic appliances, such as archwires, brackets, and miniscrews used in conventional orthodontic treatment, or attachments used in clear aligner treatment, including those taken at the end of treatment. These images present challenges for detecting gingivitis, supragingival calculus, and plaque due to metallic reflections and partial obstruction of dental and gingival surfaces, complicating both human annotation and algorithmic interpretation. Recent studies have applied preprocessing techniques, such as gamma correction and Otsu thresholding, to reduce these artifacts [9]. In future studies, applying models trained with images containing fixed orthodontic appliances may improve the clinical accuracy and comprehensiveness of periodontal assessment procedures by facilitating better adaption to different stages of orthodontic treatment.

Finally, our study used a single type of equipment to ensure standardization during imaging. However, teledentistry applications require evaluating images taken by patients with their own devices and under variable lighting conditions. Thus, real-time detection can be achieved by integrating images from mobile cameras into these systems. Therefore, in future studies, it would be beneficial to focus on advancing AI models capable of analyzing images with varying qualities and perspectives, making them more adaptable for nonclinical applications.

## Conclusion

We evaluated the YOLOv8 and U-Net + ResNet50 models to automatically detect periodontal conditions and evaluated their clinical applicability. Our results showed that both models achieve high accuracy, but the U-Net + ResNet50 model was more successful in detecting soft tissue conditions, whereas the YOLOv8 model was more successful in identifying hard tissue structures. Therefore, DL-based systems can be used as decision support tools by providing fast and objective evaluations, especially in teledentistry applications with limited clinical access. However, the final diagnostic decision should always be based on the expert judgment of the clinician, and these models should be used with caution as auxiliary tools.

## Data Availability

No datasets were generated or analysed during the current study.
